# Control of Facet Preference and Stability with Halogen Passivation of CsPbBr_3_ Perovskite

**DOI:** 10.1021/acsami.5c10877

**Published:** 2025-09-12

**Authors:** Xiangyue Cui, Hejin Yan, Hongfei Chen, Xing Liu, Yongqing Cai

**Affiliations:** Joint Key Laboratory of the Ministry of Education, Institute of Applied Physics and Materials Engineering, 59193 University of Macau, Taipa, Macau SAR 999078, China

**Keywords:** CsPbBr_3_ nanocrystals, surface energetics, halogen passivation, perovskite stability, first-principles calculations

## Abstract

Cesium lead bromide perovskite nanocrystals (CsPbX_3_, X = Cl, Br, I) have garnered significant attention due to their facile controllable synthesis and remarkable optoelectronic properties. Herein, we reveal the energetics of surfaces to account for the formation preference of surface orientations and terminations through first-principles calculations. Surface energetics, which are difficult to quantitatively measure in experiments, are the underlying thermodynamic indicators that govern the sample morphology, growth direction, and surface stability. For the first time, we establish a hierarchy of surface energy for various surfaces (named as Miller indices-terminations), ordered under the CsBr-rich condition as follows: (001)-CsBr < (100)-Br < (110)-PbBr_2_ ≈ (001)-PbBr_2_ < (100)-PbBr < (010)-CsPbBr < (100)-CsPbBr < (100)-Br_2_. Hence, the lowest CsBr-terminated (001) surface tends to be the most popular and surviving one in CsPbBr_3_ nanocrystals. Interestingly, adoption of appropriate halogen atoms (F, Cl, and I) as adsorbents can lead to a reversal in the trend of surface energies. This allows intentional control of the popularity of certain surface indexes with enhanced performance. Our work presents an atomic-scale mechanism and proposes an effective strategy for improving the surface stability of CsPbBr_3_, which is crucial for guiding experimentalists on designing more efficient and stable perovskite nanocrystals.

## Graphical abstract

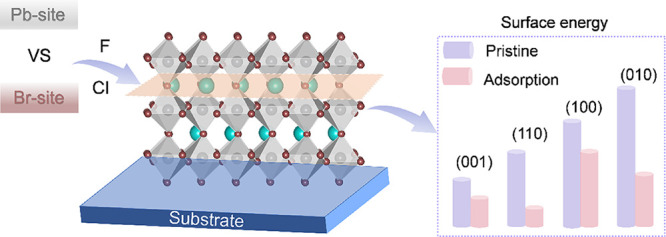

## 1 Introduction

All-inorganic metal halide perovskites (MHPs) have received substantial attention due to their strong light absorption,^1,2^ long carrier diffusion,^2,3^ tunable bandgap, and low manufacturing cost,^4,5^ making them possess applications in the fields of solar cells,^6,7^ photocatalysis,^8^ photodetectors (PDs),^9^ light-emitting diodes (LEDs),^10^ lasers,^11^ etc. Compared with traditional semiconductors, chemical structures of all-inorganic MHPs are more diverse due to a wide range of compositions and dimensions. The CsPbX_3_ (X = Cl, Br, I) perovskites, a representative family of MHPs, have been fabricated into various nanoscale structures, including zero-dimensional (0D) quantum dots,^12–14^ one-dimensional (1D) nanowires,^15,16^ and two-dimensional (2D) nanoplates.^17^ The relationship between nanocrystal (NC) structures and their properties, however, was difficult to extract, until the visualization of the atomic structure of ultrathin 2D CsPbBr_3_ was first achieved by using low dose-rate in-line holography,^18^ which allows a quantitative evaluation of lattice distortion. Moreover, the multilayer diffraction technique can accurately measure the thickness, crystal structure, stoichiometry, coverage, and even surface passivation type of ultrathin nanoplates,^19^ all of which are crucial for characterization of the surface of 2D perovskites.

Due to their inherent ionic nature, CsPbBr_3_ NCs exhibit poor stability under ambient conditions and surface reconstruction occurs,^20,21^ which severely hampers their practical deployment. To solve this issue, various strategies were proposed to passivate the surfaces of the NCs. For instance, to heal surface trap states in CsPbBr_3_ NCs, a combined treatment with didodecyldimethylammonium bromide and lead bromide was adopted,^22^ generating robust colloids with high purity and high photoluminescence quantum yield. The addition of extra oleylammonium bromide as ligand synthesized CsPbBr_3_ NCs, via passivating the surface Br^–^ ions, improves colloidal durability and retains green emission with increased photoluminescence (PL) intensity.^20^ By using short-chain ligands, such as benzoic acid (BA) and ascorbic acid (AA), instead of the more commonly used oleic acid (OA) and oleylamine (OLA) for post-treatment of the perovskite NCs, the stability and optical properties were enhanced, concomitantly overcoming the charge transport limitations associated with the insulating nature of longer chain ligands.^23^ Active learning was adopted to accelerate the identification of ideal ligands by screening over 160,000 organic molecules.^24^ Actually, there are a series of similar works, all involving pairing of anions and cations between the surface and organic capping ligand.^25–30^ The use of NH_4_SCN as an additive in the precursor solution enables the preparation of high-quality CsPbBr_3_ films with reduced trap state density and effectively suppresses interfacial carrier recombination.^31^

In thin films and semiconductor systems, surface and interfaces are pervasive because they offer additional flexibility to modulate the properties of devices.^32^ Several studies have reported that quasi-2D perovskites present better stability than their 3D bulk counterparts.^33,34^ The stability of the surface structure is governed by surface energy, which represents the energy cost for a surface cleaving from an infinite solid. For CsPbI_3_, the thermodynamic stability of orthorhombic γ-CsPbI_3_ is preferred over that of δ-CsPbI_3_ as the former has lower surface energy.^35^ Among (100), (110), and (111) surfaces, the nonpolar (100) surface of cubic CsSnX_3_ (X = Cl, Br, I) is more stable than the others, and this preference is independent of the types of X.^36^ Intrinsically, the stability of surface structures in MHPs is governed by multiple factors, including surface dipoles and work functions,^37^ surface defects and passivation effect,^38–40^ ion kinetics,^41^ and even surface phonons.^42^

CsPbBr_3_ perovskite undergoes multiple temperature-dependent structural phase transitions from high-temperature cubic (α-phase) to tetragonal (β-phase) to orthorhombic (γ-phase, room-temperature phase) at relatively low temperatures.^43,44^ Compared to other members of the all-inorganic perovskite NC family, such as CsPbCl_3_ and CsPbI_3_, CsPbBr_3_ NCs exhibit fewer Br vacancies and possess optimal tolerance factors.^45^ To date, numerous studies have focused on the surface properties of cubic NCs,^46–49^ while the orthorhombic phase is overlooked. Although the (001) facet of γ-CsPbBr_3_ has proven to exhibit the lowest surface energy,^20,50^ identifying ways to stabilize other facets is highly appealing as other surfaces may be more superior in terms of optoelectronic performance. Here, we systematically explore the impact of surface orientations and terminations on the stability and electrical properties of orthorhombic CsPbBr_3_ by first-principles calculations. Among all of the pristine surfaces considered, the CsBr-terminated (001) surface possesses the lowest surface energy, followed by the PbBr_2_-terminated (110) surface. When appropriate halogen atoms are introduced for adsorption at the topmost termination, the order of surface preference alters and the stability of these surfaces is significantly enhanced, as evidenced by a decrease in the surface energy, particularly for the CsPbBr-terminated (010) surface. Generation of rare surface facets like (100), (010), and (110) in orthorhombic CsPbBr_3_ can be useful to control the directionally of light emission or absorption, which is valuable for applications such as directional lighting in perovskite LEDs or polarized light detection in PDs due to their distinct surface energies, atomic coordination environments, and termination-dependent chemical activities. Therefore, our study provides atomic-scale insights into the interaction between adsorbed atoms and pristine surfaces, offering valuable reference for improving the stability and tuning the morphology of MHP NCs.

## 2 Computational Methods

Our calculations were performed based on the spin-polarized density functional theory (DFT) and implemented in the Vienna ab initio simulation package.^51^ The projected augmented wave pseudopotentials and generalized-gradient approximation functional with the van der Waals correction with the DFT-D3 method^52^ were adopted as the exchange-correlation functional.^53^ The plane-wave basis with kinetic energy was set at 400 eV, and the atomic positions of the slabs were partially relaxed until the residual Hellmann–Feynman forces on each atom were less than 0.02 eV/Å. We generated four low-index surfaces and used the k-point grids with Γ-centered Monkhorst–Pack of 6 × 6 × 1, 6 × 4 × 1, 4 × 6 × 1, and 4 × 4 × 1 for (001), (100), (010), and (110) facets. A vacuum layer of 15 Å was chosen for symmetric slabs to reduce the interaction between the surfaces.

By including effects of both bond cleaving (σ^cl^) and surface relaxation (σ^relax^), the surface energy (σ) can be defined as follows:

$$\sigma^{\mathrm{cl}}=\frac{E_{\mathrm{slab}}^{\mathrm{unrel}}-E_{\mathrm{bulk}}+\underset{i}{\sum }n_{i}\mu_{i}}{2S}$$ 1

$$\sigma^{\mathrm{relax}}=\frac{E_{\mathrm{slab}}^{\mathrm{rel}}-E_{\mathrm{slab}}^{\mathrm{unrel}}}{S}$$ 2

$$\sigma =\sigma^{\mathrm{cl}}+\sigma^{\mathrm{relax}}$$ 3

where *σ* ^cl^ is the cleavage energy when forming the unrelaxed slab and *σ* ^relax^ is the change of surface energy caused by optimization. In the calculation, we fixed a few bottom layers of atoms on the symmetric surface to simulate the bulk phase; therefore, there are two exposed surfaces during dissociation, and a factor of 1/2 appears in eq 1. The $E_{\mathrm{slab}}^{\mathrm{unrel}}$ is the energy of the unrelaxed slab, and *E*_bulk_ is the total energy of bulk formula units in the surface. The term ∑*n*_*i*_μ_*i*_ is to account for the excess number (*n*_*i*_) of species *i* and the energy of the nonstoichiometric surface as a function of the chemical potentials (μ_*i*_) of the constituent elements, which are relative to the total energy of each constituent element *E*_*i*_. The $E_{\mathrm{slab}}^{\mathrm{rel}}$ is the energy of relaxed slabs, and *S* in the equations is the surface area. More detailed calculations are presented in the Supporting Information (SI).

For halogen atoms (F, Cl, and I) and the molecule SCN adsorbing on these surfaces, they are merely bound to the topmost layer of each surface, and the number of fixed atomic layers is exposed and consistent with that of the clean surfaces. To rigorously compare the energies with different terminations after adsorption, we employ a high surface coverage of 100% on both the exposed Br- and Pb-site. Among them, we tried two vertical binding formats for molecular adsorption to identify the most stable configuration. For SCN, both downward and upward relative alignments with the surfaces are considered. The adsorption energy (*E*_ads_) is calculated as

$$E_{\mathrm{ads}}=E_{\mathrm{total}}-E_{\mathrm{surface}}-E_{\mathrm{atoms}/\mathrm{mol}}$$ 4

where *E*_total_, *E*_surface_, and *E*_atoms/mol_ are the energies of atoms or molecules of the adsorbed CsPbBr_3_ system, pristine surface, and adsorbents (atoms or molecules), respectively. (atoms or molecules), respectively.

## 3 Results and Discussion

Among the three phases of bulk CsPbBr_3_ perovskite, the orthorhombic phase with a tilted octahedral network is the most stable structure at room temperature. The equilibrium lattice parameters are *a* = 8.26 Å, *b* = 8.49 Å, *c* = 11.89 Å (α = β = γ = 90°), which are in fairly good agreement with previous reports.^54,55^ However, the phase stability of CsPbBr_3_ perovskite nanocrystals (PNCs) exhibits a size effect, and there are ongoing debates regarding whether the actual crystal structure is orthorhombic or cubic. Brinck et al. have corroborated that the PNCs crystalline core exhibits an orthorhombic structure,^56^ and recent theoretical calculations also confirmed the rationality of choosing the orthorhombic structure as the initial phase to study the surface properties of CsPbX_3_ perovskites.^50^ To this end, we constructed four representative surfaces, namely, (001), (100), (010), and (110), along several low-index directions by carving the relaxed orthorhombic bulk phase (space group *Pbnm*, No. 62), and their crystal configurations are illustrated in Figure 1. We first cleaved the bulk along the <100> direction to produce terminals such as Br, Br_2_, PbBr, and CsPbBr, creating a series of surfaces designated as (100)-Br, (100)-Br_2_, (100)-PbBr, and (100)-CsPbBr, respectively (Figure 1a–d). Similarly, two surfaces terminated by the CsBr layer and the PbBr_2_ layer, denoted as (001)-CsBr and (001)-PbBr_2_, respectively, are obtained, with their normal aligned along the <001> direction (Figure 1e,f). These surfaces consist of alternating CsBr and PbBr_2_ layers stacked together. There is evidence to suggest that the (100) and (010) surfaces have similar in-plane lattice constants.^57^ Thus, to compare the effect of surface orientation on relative stability, we selected CsPbBr and PbBr_2_ terminals along the (010) and (110) directions, respectively, as shown in Figure 1g,h, respectively.

**Figure 1:**
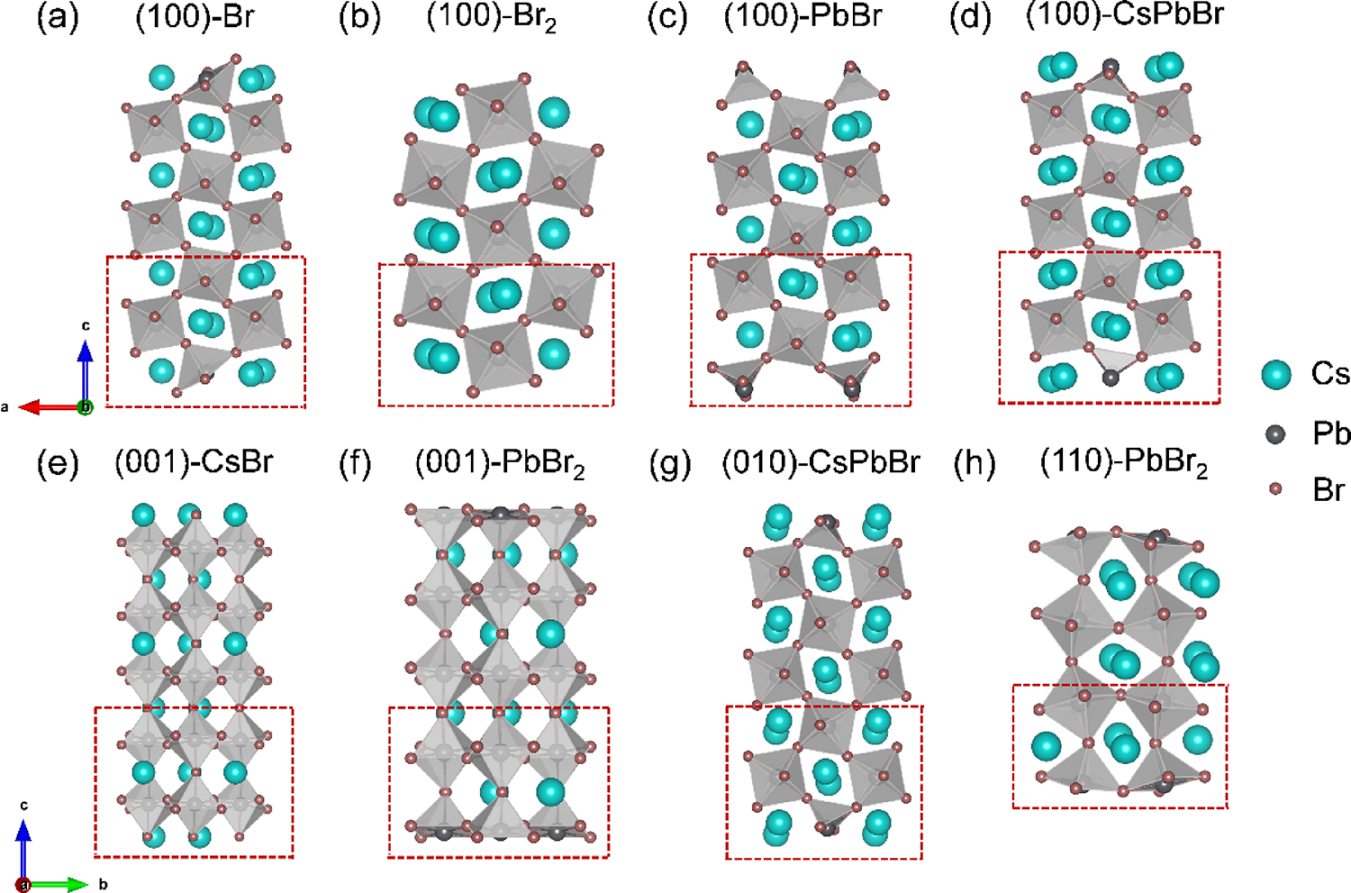
Unrelaxed bare slab models of orthorhombic CsPbBr_3_. (a–d) The (100) surfaces with Br-, Br_2_-, PbBr-, and CsPbBr-termination, respectively. (e, f) The (001) surfaces with CsBr- and PbBr_2_-termination. (g) The (010) surface with CsPbBr-termination, and (h) (110) surface with PbBr_2_-termination. The bottom layers were fixed at their bulk positions with red dashed rectangles, where cyan, dark gray, and brown spheres represent Cs, Pb, and Br atoms, respectively.

To evaluate and compare the energy cost of forming different surfaces in CsPbBr_3_, we calculate the *σ* of common low-index pristine surfaces with different surface terminations, as shown in Figure 2. Among them, the (001) surface with CsBr-termination exhibits the lowest *σ* value (0.138 and 0.020 J/m^2^, under both CsBr-rich and PbBr_2_-rich growth conditions, respectively), regardless of the growth conditions. A lower *σ* means less energy cost and a more stable surface, implying that the (001) surface can survive among other surfaces during the growth of the CsPbBr_3_ nanocrystal or nanoclusters. In contrast, the PbBr_2_-terminated (001) one is less stable due to the high ratio of exposed Pb cations on the surface. For the (100) surfaces, the *σ* of Br-termination is independent of chemical potentials and possesses the highest stability with the lowest *σ* of 0.218 J/m^2^ among various terminations. This exceptional stability stems from the Br-termination being the only stoichiometric termination, which maintains the bulk CsPbBr_3_ composition intrinsically without requiring additional atoms or vacancies. In contrast, the CsPbBr- and Br_2_-terminated surfaces are less stable and more sensitive to the growth environment. Under CsBr-rich and PbBr_2_-rich conditions, the *σ* values of the CsPbBr-termination (100) surface are 0.657 and 0.768 J/m^2^, respectively, while those of the Br_2_-terminated surface are 0.750 and 0.675 J/m^2^.

**Figure 2:**
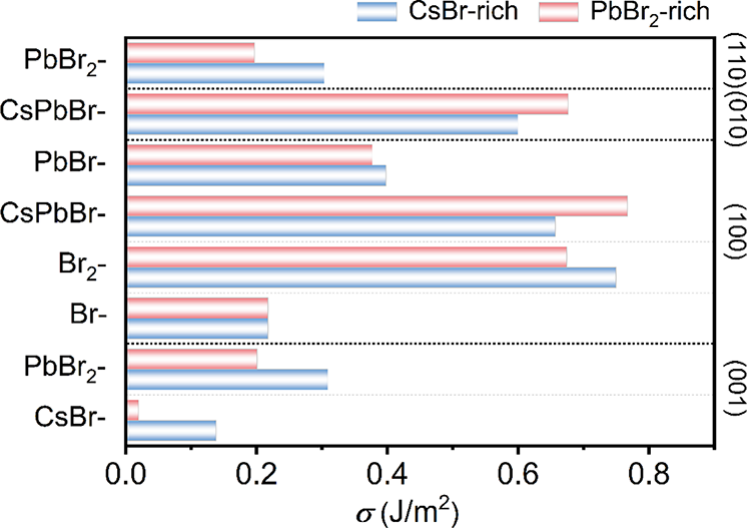
Comparison of the surface energy *σ* of four pristine facets with different terminations of orthorhombic CsPbBr_3_ under both CsBr-rich and PbBr_2_-rich growth conditions. The Pb-rich growth condition (Δ*μ*_Pb_ = 0) is considered in all cases.

We next analyze the relationship between local structural features and surface stability. For surfaces with the same CsPbBr-termination, the (010) surface exhibits slightly better stability than the (100) surface. This discrepancy is related to the formation of the Pb–Pb dimer in the (100) surface after full relaxation, which significantly disrupts the original octahedral vertex connection and reduces the coordination number of Pb. This leads to lattice distortion in the outermost surface layer, as the strength of the Pb–Pb bond is considerably weaker than that of the Pb–Br bond. For PbBr_2_-termination, the difference in *σ* between (110) and (001) surfaces is negligible. This similarity arises from the fact that both surfaces exhibit symmetric octahedral tilting of PbBr_6_ polyhedra, with each Pb^2+^ ion coordinated by five Br^–^ ions in a geometrically symmetric pattern. In contrast, the (100)-Br_2_ facet (Figure 1b) undergoes excessive Br^–^ accumulation, inducing severe Pb–Br bond distortion, that is, Pb^2+^ coordinates with Br^–^ ions in asymmetric bond lengths (partial bond elongation or compression). Therefore, the stability of the surface can be intuitively reflected by changes in the local configuration, which we will discuss in detail in the following sections.

In short, based on the calculated *σ*, we predict the general trend of the relative stability of various surfaces in CsPbBr_3_ under CsBr-rich conditions as follows: (001)-CsBr < (100)-Br < (110)-PbBr_2_ ≈ (001)-PbBr_2_ < (100)-PbBr < (010)-CsPbBr < (100)-CsPbBr < (100)-Br_2_. We also calculated the defect formation energy of the surfaces (Figure S1), revealing a similar trend as *σ*. Our results indicate a generally more stable and high popularity of the (001) and (100) surfaces, which is consistent with that of the experimental observation, showing the existence of (001) and (100) surfaces in CsPbBr_3_ NCs.^56^ Notably, the PbBr_2_-terminated (110) and (001) surfaces possess comparable *σ*, which correlates with experimental high-resolution transmission electron microscopy (HRTEM) observations showing (110) and (002) crystal planes with a small plane *d*-spacing difference.^9^ This comparable energetics rationalizes why both facets can coexist in multidirectional coupling during nanorod formation, as their comparable thermodynamic stability allows for flexible orientation in growth. In comparison, the *σ* in CsPbBr_3_ is generally much lower than those of perovskite oxides such as CaTiO_3_, SrTiO_3_, and BaTiO_3_ ^58–61^ because of the soft nature of all-inorganic MHPs with a low binding strength. The *σ* of the cubic phase is generally lower than the corresponding orthorhombic phase due to its low surface-to-volume ratio without any octahedral inclination.^50^

As expected, we find surface reconstruction or relaxation, the degree of which varies with the different terminated surfaces. This can be quantified by surface rumpling (*s*), defined as the relative displacement of anion and cation species in the surface, which is commonly discussed in the study of oxide perovskites.^53,61,62^ Here, we examine and compare the *s*, determined by the relative vertical displacement of Br atoms with respect to the metal atoms (Cs or Pb) in the topmost surface layer. This displacement is calculated as *s* = *Z*_Br_ – *Z*_M_, where *Z*_Br_ and *Z*_M_ represent the average vertical positions (*z*-coordinates) of Br anions and metal cations (Cs/Pb) in the topmost layer, respectively. For clarity, a schematic diagram is shown in Figure 3a. For all eight surfaces of CsPbBr_3_, it is found that there is negative and nearly zero rumpling of PbBr_2_-termination for all of the surfaces, indicating a good conservation of the Pb–Br equatorial plane on the surface. Considering that merely Br atoms are exposed on the (100) surface, the variation of rumpling is defined on the first sublayer. The Br_2_-terminated surface shows a larger outward displacement (toward vacuum) on average than that of the Br-terminated surface, with the *s* of Br_2_ termination being 0.234 Å larger than that of the latter. On the other hand, both the CsPbBr-terminated (100) and (010) surfaces exhibit relatively large and positive *s* values, indicating significant outward displacement of Br atoms in the topmost layer. The significant *s* values explain the poorest stability of such CsPbBr-terminated surfaces. In contrast, the (001) surface has the lowest *σ* value, probably due to the formally neutral CsBr and PbBr_2_ terminals. However, the absolute value of *s* at the CsBr-termination is larger than that of PbBr_2_, which can be attributed to the large ionic radius of Cs^+^ and the weaker bond strength of Cs–Br compared to that of Pb–Br. Consequently, the CsBr-termination is prone to displacement at the surface, resulting in a relatively large rumpling amplitude. A similar discussion holds for PbBr-terminated in the (100) surface, accounting for its small rumpling. Overall, the minimal rumpling amplitude |*s*| correlates strongly with higher stability.

**Figure 3:**
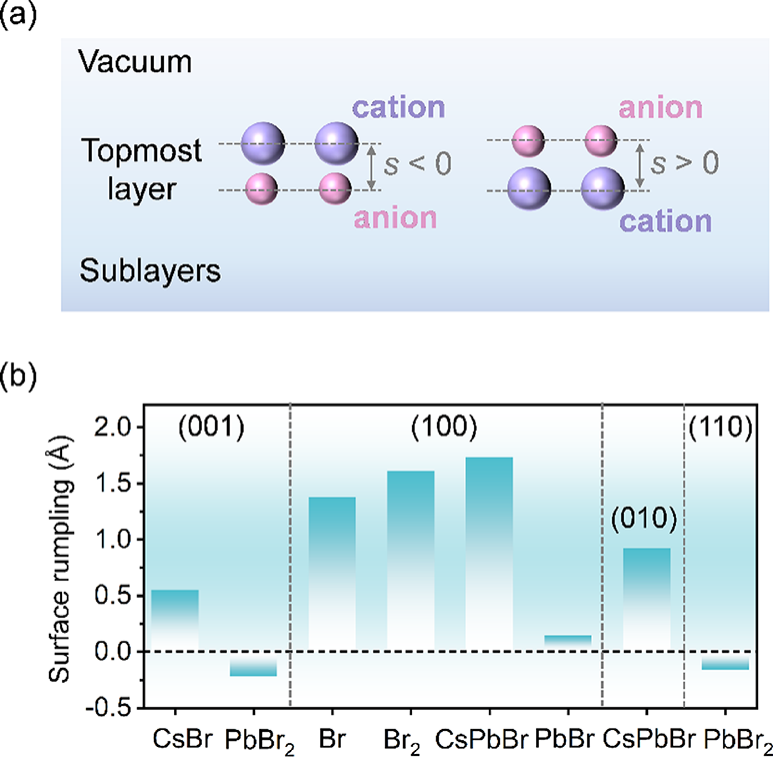
(a) Schematic diagram of surface rumpling (*s*) on the topmost layer, the values of which are defined as negative (positive) for anions (cations) closer to the vacuum region. (b) Surface rumpling of CsPbBr_3_ surfaces with different terminations. Note that for the (100) surface with Br- and Br_2_-termination, the rumpling is analyzed with the first sublayer instead as only Br atoms appear in the topmost layer.

We further calculate the local density of states (LDOS) of all considered surfaces and the total density of states (TDOS) of the bulk CsPbBr_3_ for comparison. The band edge states are primarily composed of orbitals of Pb and Br atoms, whereas Cs contributes negligibly to the states, although it can affect the electronic structure indirectly by tilting the [PbBr_6_]^4–^ octahedra. In the cases of the (100) and (001) surfaces (Figure 4a,b), their LDOS largely maintain electronic characteristics as the TDOS of bulk CsPbBr_3_ (gray short dotted lines) regardless of surface terminations, except for the CsPbBr-terminated (100) surface, which introduces trap states near the Fermi level (*E*_F_). The trap states originate from the outermost surface layer of the CsPbBr-terminated structure undergoing reconstruction after structural relaxation, resulting in changes in the coordination environment around Pb and the formation of a Pb–Pb dimer (2.94 Å). This behavior reflects the presence of dangling states, leading to the instability of the CsPbBr terminal on the (100) surface, and corroborates our hypothesis in analyzing surface rumpling in Figure 3b. For the CsPbBr-terminated (010) surface, the distortion of the surface octahedral leads to a redistribution of electronic states, causing the *E*_F_ to move upward into the conduction band region as illustrated in Figure 4c. Notably, for the Br_2_-terminated (100) surface, the topmost two Br atoms are strongly reconstructed and form bonds with the sublayer Pb, with a bond length of 2.74 Å, close to the bulk Pb–Br length of 3.04 Å. Therefore, the Br_2_-terminated (100) surface maintains the bonding character of bulk CsPbBr_3_, but with a surface-specific stoichiometry deviation, namely, an excess of Br. To balance this, electrons are partially transferred from Pb to Br atoms, inducing a Br-dominated surface state near the *E*_F_, which acts as an acceptor-like trap. Thus, the LDOS of this surface exhibits a p-type behavior. In a word, for all of the terminations except for the CsPbBr case, the surfaces largely maintain the characteristics of bulk DOS, indicating a good surface-tolerant nature of CsPbBr_3_ in terms of electronic structure.

**Figure 4:**
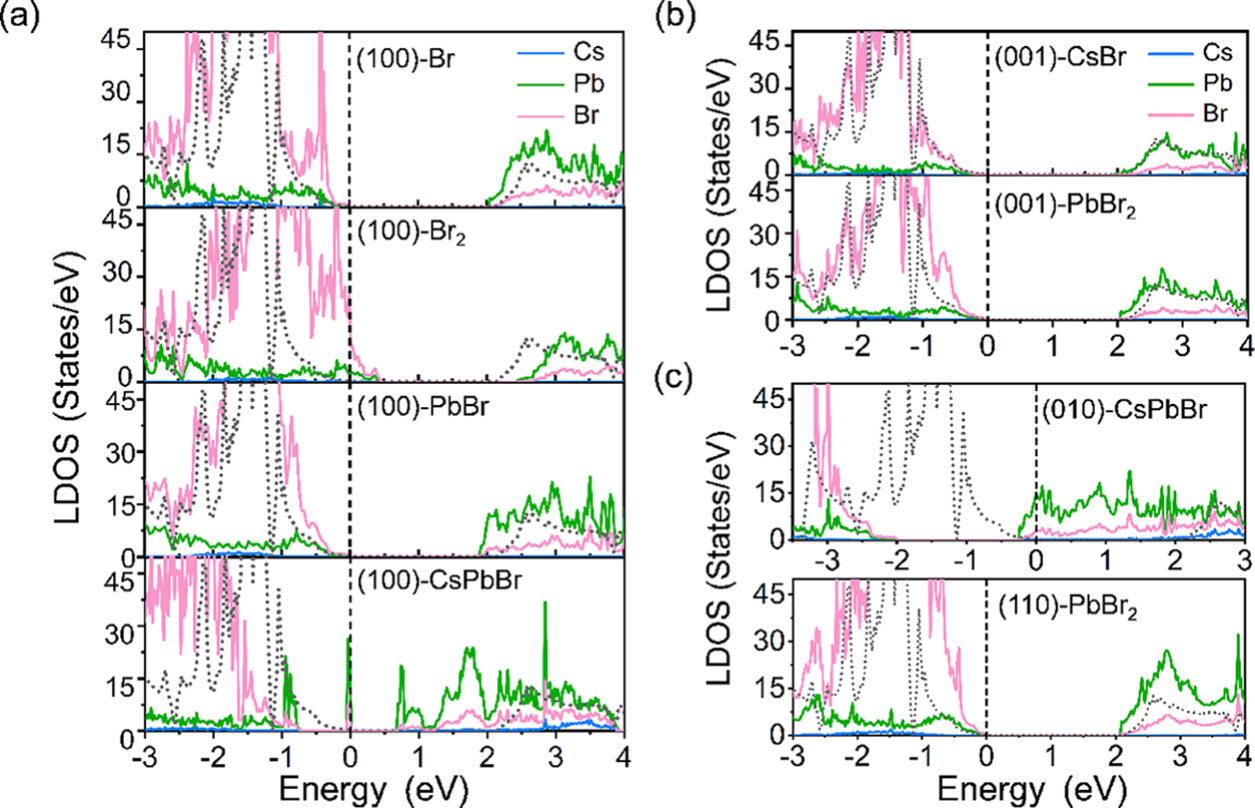
LDOS of CsPbBr_3_ slabs with different terminations along several low-index surfaces. (a) (100) surface, (b) (001) surface, and (c) (010) and (110) surfaces. The gray, short dotted lines in all figures represent the TDOS of the bulk CsPbBr_3_ for comparison, and the black dashed lines near zero are the position of the *E*_F_.

For CsPbBr_3_, one of the major factors limiting its potential applications is the inevitable formation of defects, particularly the emergence of Br vacancy clusters in the bulk^63^ due to extensive radiation and thermal excitation. Given the impact of intrinsic defects on the stability and performance of the material, we explore the possibility of tuning the intrinsic stability of the CsPbBr_3_ surfaces through surface decoration. This approach could potentially mitigate the effects of defects by synthesizing NCs with certain facets and may alter the surface order in terms of *σ*. We then conducted a systematic study through the adsorption of three halogen elements (F, Cl, and I) on various surfaces, with the corresponding adsorption energies being compiled in Table 1. For surfaces with both Pb and Br atoms exposed at the topmost layer, these halogen atoms tend to preferably adsorb at the Pb-site with lower *E*_ads_.

**Table 1:** Adsorption Energies *E*_ads_ (eV) of Halogen Species (F, Cl, and I) on Various Surfaces of CsPbBr_3_

		adsorbate atoms		
surface	termination	F	Cl	I
(001)	CsBr	–0.548	1.097	1.036
	PbBr_2_	–0.712 (−2.799)	2.245 (−0.088)	2.045 (0.602)
(100)	Br	0.322	1.928	1.729
	Br_2_	–1.892	1.169	0.807
	PbBr	–0.505 (−2.801)	1.285 (−0.039)	1.416 (0.684)
(010)	CsPbBr	–4.464 (−8.235)	–3.621 (−5.439)	–3.465 (−4.443)
(110)	PbBr_2_	–1.406 (−5.616)	4.486 (−0.202)	3.069 (1.167)

The calculated *σ* values of CsPbBr_3_ surfaces with different adsorbates (F, Cl, I, S, C, and N species) are shown in Figure 5. Compared to clean surfaces, the termination with F atoms yields the lowest σ values for all surfaces. Considering the similarity between CsPbBr_3_ and FAPbI_3_, our results may explain the recent experiment by Zhao et al., reporting that vapor-phase fluoride treatment on the surface of FAPbI_3_ samples suppresses defect formation and ion diffusion, thereby enhancing the stability of PSCs.^64^ Almost all surfaces with specific atomic adsorption, regardless of growth conditions, are more stable than the corresponding pristine surfaces, except for the Br-terminated (100) surface (Figure 5c).

**Figure 5:**
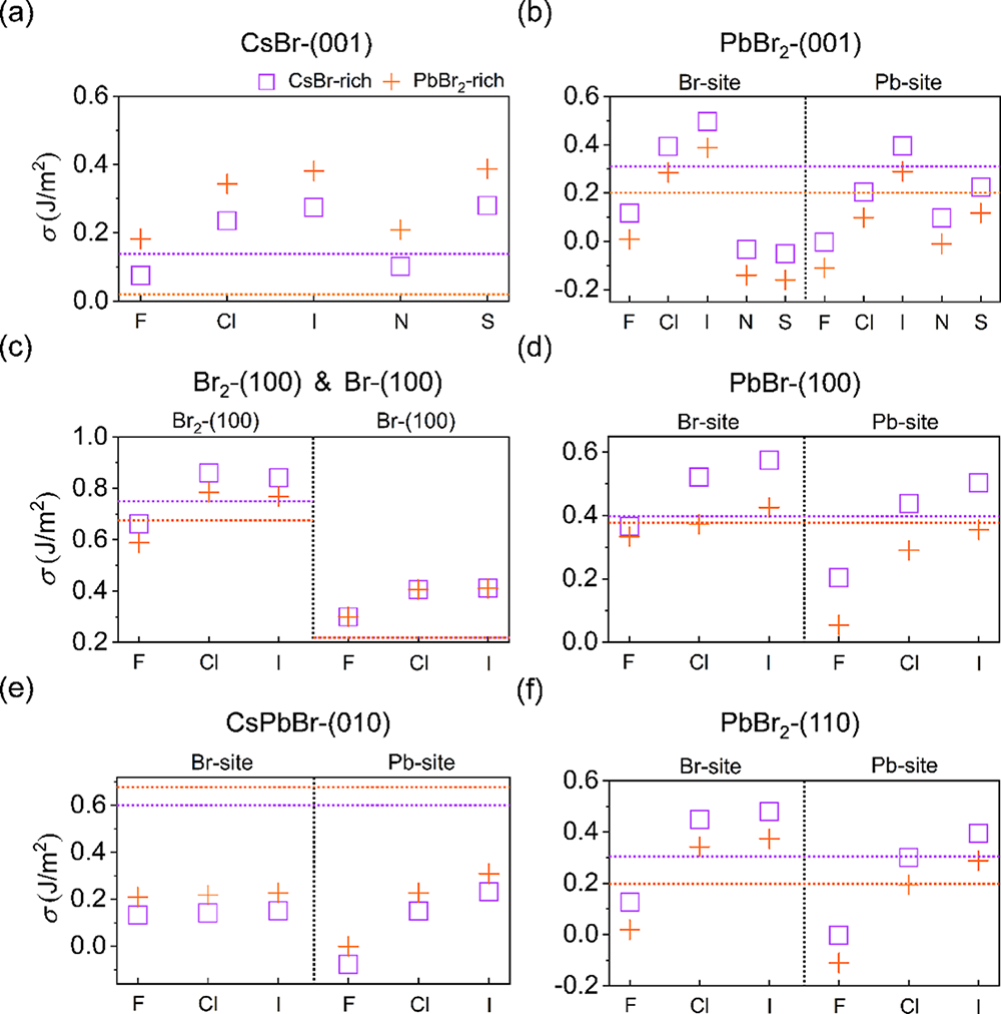
Effect of the adsorption of halogen species (F, Cl, and I) on modulating the surface energy *σ* of orthorhombic CsPbBr_3_. (a) CsBr- and (b) PbBr_2_-terminated (001) surfaces. Br_2_-,Br- and PbBr-terminated (100) surfaces are presented in panels (c) and (d), respectively. (e) CsPbBr-terminated (010) surface. (f) PbBr_2_-terminated (110) surface. The lilac squares and orange crosses indicate growth conditions that are CsBr-rich and PbBr_2_-rich, respectively. The lilac and orange dotted horizontal lines denote the *σ* values of clean surfaces under the corresponding growth conditions.

Interestingly, we found that an appropriate adsorbate can reverse the order of surface energy. Contrary to the pristine case, the *σ* of the PbBr_2_-terminated (001) surface is lower than that of the CsBr-terminated (001) surface when F and Cl atoms are absorbed at the Pb-site, as shown in Figure 5a,b. A similar behavior is also observed on the (010) surface in Figure 5e, where the *σ* values after adsorption are significantly lower than those of any termination on the (100) surface. For comparison, we select the (001) surface with intrinsically low *σ* values as representative and introduce a pseudohalide anion with −1 charge (SCN) to evaluate its effect on surface stability (refer to Figure S2 in the Supporting Information for relaxed structures). The calculated *σ* for SCN adsorbed on the PbBr_2_-terminated surface is lower than that on the CsBr-terminated surface, indicating that the molecule is more inclined to adsorb on the PbBr_2_ termination, as also reflected in *E*_ads_ (Table S1). In a word, despite the pristine (010) and (110) surfaces being less stable compared with pristine (100) and (001) surfaces, the F-decoration can reverse the trend: F-decorated (010) and (110) surfaces (Figures 5e,f and S4 in the Supporting Information for relaxed structures) are significantly stabilized, and their surface energies are comparable to or even lower than those of the F-decorated (100) and (001) surfaces. This reversal of surface order implies the effectiveness of surface decoration to control the preferred surface of the nanocrystal during synthesis.

The mechanism underlying such surface order reversal lies in adsorbates with distinct atomic sizes and electronegativities through a synergistic interplay of compatibility and charge transfer with surface terminations. Adsorbates with strong electronegativity preferentially interact with undercoordinated Pb^2+^ cations or sites with inherent charge mismatches (e.g., CsPbBr-terminated), accepting electrons from Cs or Pb and mitigating surface charge imbalance. This not only compensates for dangling bonds but also avoids excessive steric repulsion, thereby reducing surface energy more significantly on originally less stable surfaces than those of pristine ones. In contrast, for the Br-terminated (100) surface, the adsorption of halogen atoms breaks the native stoichiometric balance and thus increases the *σ* values. Therefore, the synergistic effects of the intrinsic properties of adsorbates and surface terminations, including lattice matching and effective charge distribution, serve as the core mechanism for reversing the *σ* order, enabling the modulation of nanocrystal surface preferences via targeted surface decoration.

We chose structures with reduced *σ* values after adsorption as representatives for further DOS analysis. In all cases presented in Figure 6, the band edge of the surfaces after adsorption exhibits significant change compared with the pristine surfaces of CsPbBr_3_ shown in Figure 4. All adsorptions at the Pb-site do not introduce any in-gap states, whereas in-gap states resulting from adsorbates are found in most of the Br-site adsorptions. A similar distribution of electronic states was also observed in the adsorption of the oxygen molecule on the surface of cubic CsPbBr_3_, and these interfacial states were expected to strongly affect the behavior of photoinduced carriers and carrier mobilities.^65^ For atomic adsorption on the Pb-site, the *E*_F_ of most surfaces shifts into the valence band, while that of the (010) surface moves toward the conduction band, with the predominant states of F, Cl, and I located within the valence band.

**Figure 6:**
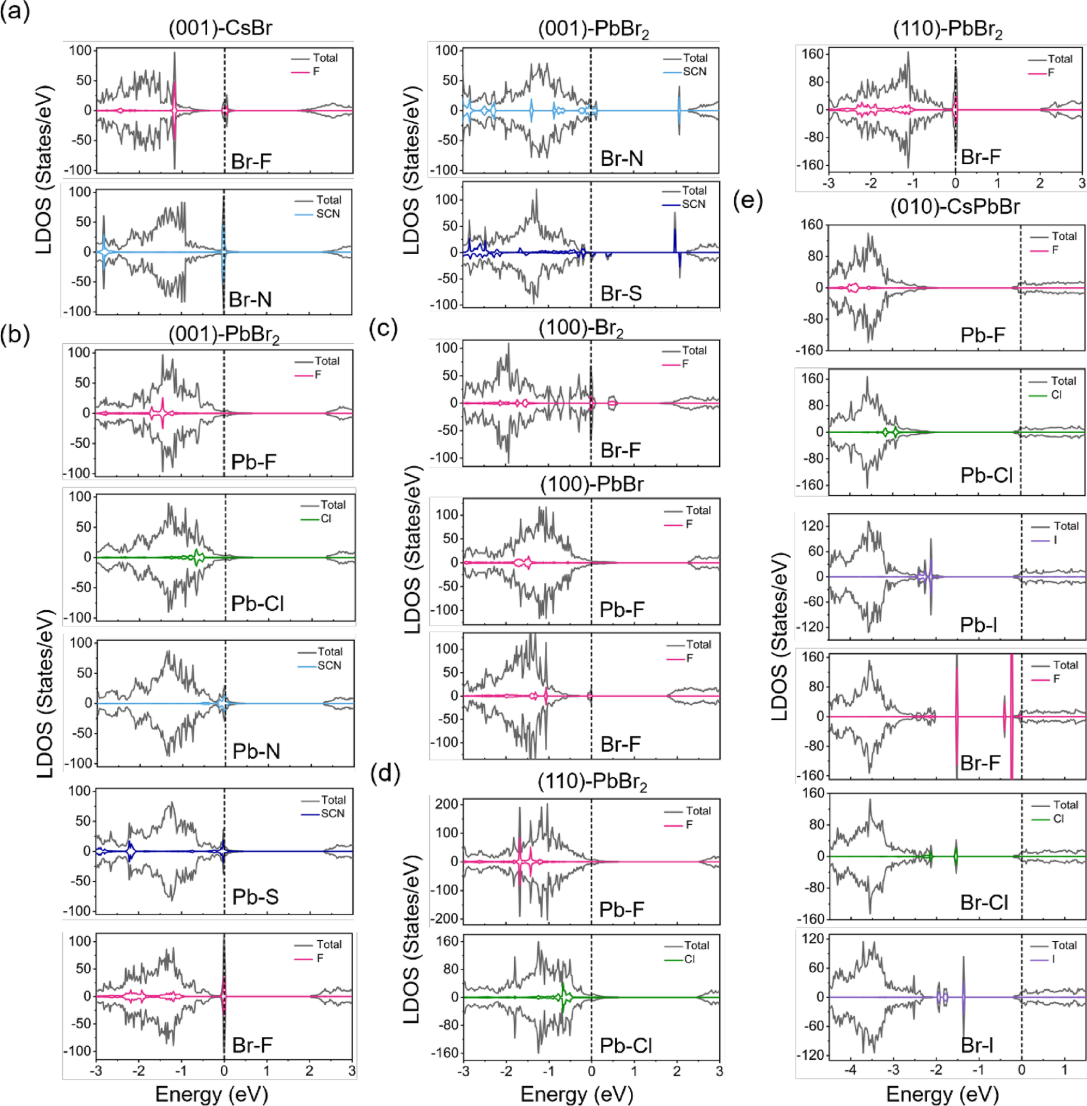
TDOS of surfaces after adsorption (dark-gray line) and LDOS of decorated CsPbBr_3_. (a,b) CsBr- and PbBr_2_-termination of the (001) surface, (c) Br_2_- and PbBr-terminated (100) surface, (d) PbBr_2_-termination of the (110) surface, and (e) CsPbBr-termination of the (010) surface. The dashed line pins the position of the *E*_F_.

To investigate the charge transfer between the adsorbates and perovskite surfaces, we calculate the differential charge density (DCD) Δρ­(*r*), which is defined as

$$\Delta \rho \left(r\right)=\Delta \rho_{\mathrm{total}}\left(r\right)-\Delta \rho_{\mathrm{surface}}\left(r\right)-\Delta \rho {\left(r\right)}_{\mathrm{ad}}$$ 5

where Δ*ρ*_total_(*r*), Δ*ρ*_surface_(*r*), and Δ*ρ*_ad_(*r*) are the charge densities of the adsorbed system, clean CsPbBr_3_ surface, and the adsorbates, respectively. For all calculations, we consider the correction of energy along the *z*-axis due to dipole–dipole interaction.^66^ The specific number of electron transfers is obtained by Bader charge analysis.^67^ In Figure 7, the total charge transferred from the CsPbBr_3_ surfaces to all adsorbed atoms is shown (with the charge obtained for each adsorbed atom presented in Figure S5). It is evident that all of the adsorbates behave as electron acceptors. Among the various adsorbates, F atoms receive the highest number of electrons from CsPbBr_3_ surfaces, ranging from +0.15 to +4.39 *e*. This phenomenon suggests a stronger interaction between F atoms and the surfaces, which can be attributed to the high electronegativity and small atomic radius of F. For the PbBr_2_-terminated (110) surface, we observe that the charge transfer of the adsorbed F atoms at the Br-site is significantly larger than that on other surfaces. This enhancement is likely due to the presence of multiple unsaturated Br sites, which provide more highly active sites for the adsorption of F atoms at 100% coverage. Similarly, the differing coordination environments of atoms on various surfaces result in variability in the activity of the adsorption sites, leading to differences in the amount of the transferred charge.

**Figure 7:**
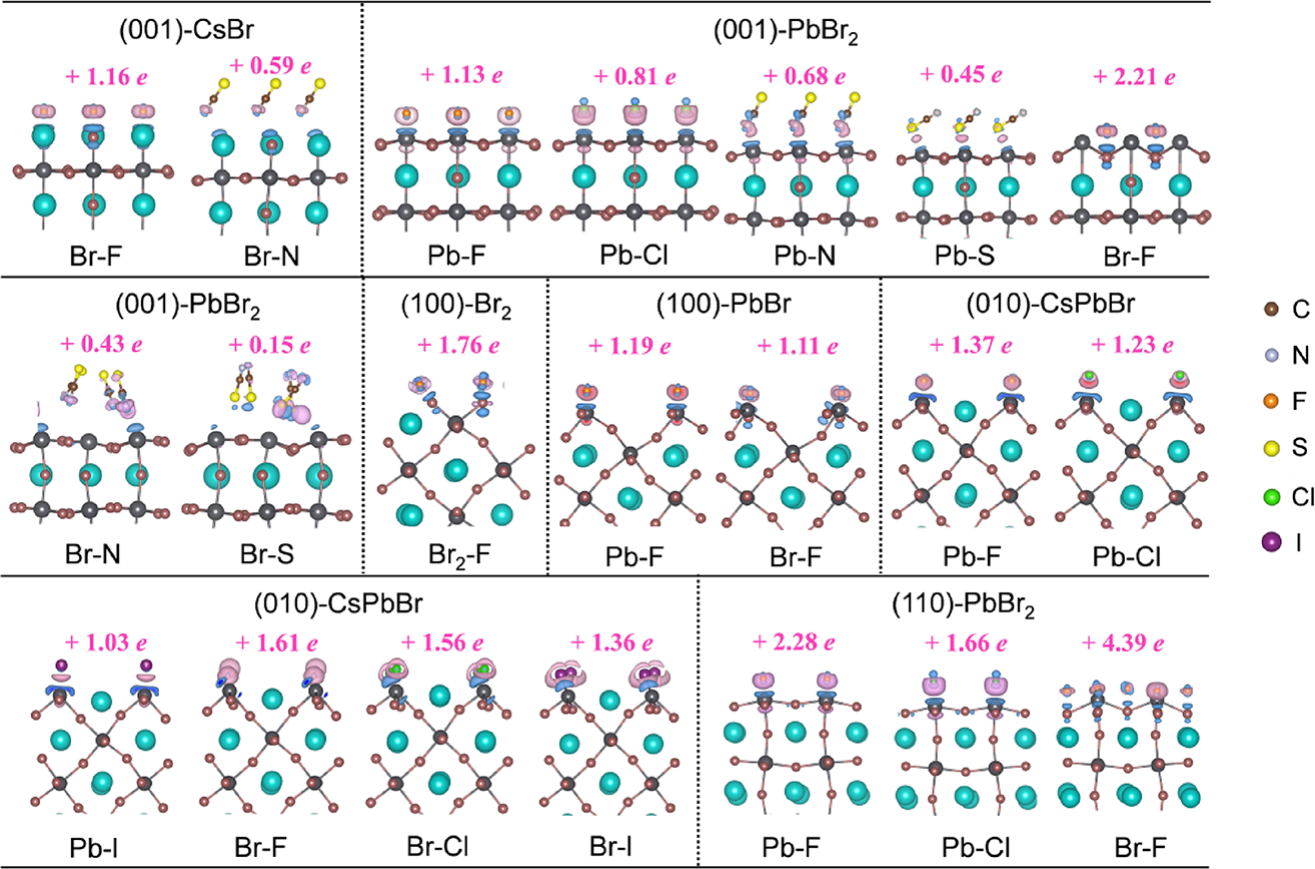
Charge density differences between adsorbates and CsPbBr_3_ surfaces, along with specific amounts of transferred charge. Pink regions denote charge accumulation, while light-blue areas signify charge depletion. The total charge transferred from the CsPbBr_3_ surface to all adsorbed atoms is shown above the top of each model.

## 4 Conclusions

In summary, we demonstrate a feasible way of evaluating and altering the order of surface energetics of the CsPbBr_3_ perovskite. We establish the following order of *σ* for clean surfaces at the CsBr-rich condition as (001)-CsBr < (100)-Br < (110)-PbBr_2_ ≈ (001)-PbBr_2_ < (100)-PbBr < (010)-CsPbBr < (100)-CsPbBr < (100)-Br_2_. Under the PbBr_2_-rich condition, the (001)-CsBr surface possesses the lowest *σ* value, followed by two energetically similar PbBr_2_-terminated surfaces, both of which have lower energies than the (100)-Br surface. This hierarchy offers insights into the growth and morphology of perovskite nanostructures. Interestingly, the selection of an appropriate halogen atom or pseudohalide as an adsorbate can induce reversal in surface stability. In particular, fluorinated surfaces possess significantly reduced *σ* values. Our work introduces novel strategies for perovskite surface engineering, enabling the precise control of surface order and the creation of rare crystallographic facets that achieve high performance.

## Supplementary Material



## Notes

Calculation method of surface energy; adsorption energies of the SCN molecule; defect formation energy of CsPbBr_3_ surfaces; and relaxed atomic configurations of (001), (100), (010), and (110) surfaces of the CsPbBr_3_ perovskite after adsorption (PDF)
