# Supplementary material for: Control of Facet Preference and Stability with Halogen Passivation of CsPbBr3 Perovskite
Source: ACS Appl Mater Interfaces. 2025 Sep 12;17(38):54190–9. doi: 10.1021/acsami.5c10877 (PMC12464902; doi:10.1021/acsami.5c10877)
Supplement: Supplementary file 1 [file am5c10877_si_001.pdf]

Supporting Information

Control of Facet Preference and Stability with Halogen Passivation of $CsPbBr_3$ Perovskite

Xiangyue Cui, Hejin Yan, Hongfei Chen, Xing Liu, Yongqing Cai*

Joint Key Laboratory of the Ministry of Education, Institute of Applied Physics and Materials Engineering, University of Macau, Taipa, Macau SAR 999078, China

*Corresponding author.
E-mail: yongqingcai@um.edu.mo (Yongqing Cai)

Calculation method of surface energy (σ)

The allowed chemical potential of each element (μ_i) when calculating σ is determined by avoiding their formation as follows:

$$\Delta H_f(\mathrm{CsPbBr_3}) = E(\mathrm{CsPbBr_3}) - E(\mathrm{Cs}) - E(\mathrm{Pb}) - \frac{3}{2}E(\mathrm{Br_2}) \quad (1)$$

$$\Delta H_f(\mathrm{CsBr}) = E(\mathrm{CsBr}) - E(\mathrm{Cs}) - \frac{1}{2}E(\mathrm{Br_2}) \quad (2)$$

$$\Delta H_f(\mathrm{PbBr_2}) = E(\mathrm{PbBr_2}) - E(\mathrm{Pb}) - E(\mathrm{Br_2}) \quad (3)$$

where E(Cs), E(Pb) and $E(Br_2)$ are total energy per atom of elemental bulk Cs, Pb and Br_2 molecule, respectively.

The μ_i under CsBr-rich condition are obtained from the formation energy of CsBr compound, as shown below, by subtracting equations (4) and (5):

$$\Delta\mu_{\mathrm{Cs}} + \Delta\mu_{\mathrm{Br}} = \Delta H_f(\mathrm{CsBr}) = -4.047\,\mathrm{eV} \quad (4)$$

$$\Delta\mu_{\mathrm{Cs}} + \Delta\mu_{\mathrm{Pb}} + 3\Delta\mu_{Br} = \Delta H_f(\mathrm{CsPbBr_3}) = -7.855\,\mathrm{eV} \quad (5)$$

Similarly, the μ_i under $PbBr_2$-rich condition can be obtained from the formation energy of $PbBr_2$ compound by subtracting equations (5) and (6)

$$\Delta\mu_{\mathrm{Pb}} + 2\Delta\mu_{\mathrm{Br}} = \Delta H_f(\mathrm{PbBr_2}) = -3.339\,\mathrm{eV} \quad (6)$$

Table S1 Adsorption energies E_{ads} (eV) of SCN molecule on the (001) surface of $CsPbBr_3$ at the Br-site and Pb-site

Termination	Br-site		Pb-site	
	Br-N	Br-S	Pb-N	Pb-S
CsBr	-0.761	-0.389	-	-
$PbBr_2$	-2.882	-2.736	-1.599	-1.007

Defect formation energy for intrinsic $CsPbBr_3$ surfaces using the following formula:

$$\Delta H(\alpha) = E(\alpha) - E(host) + \sum_i n_i (E_i + \mu_i) \quad (7)$$

where E (α) is the total energy of a surface, E(host) the total energy of bulk $CsPbBr_3$. The integers n_i is the number of atom i that added ($n_i < 0$) or removed ($n_i > 0$) from the host structure to form a defective one. E_i is the total energy per atom of constituent elemental and μ_i is the corresponding atomic chemical potential.

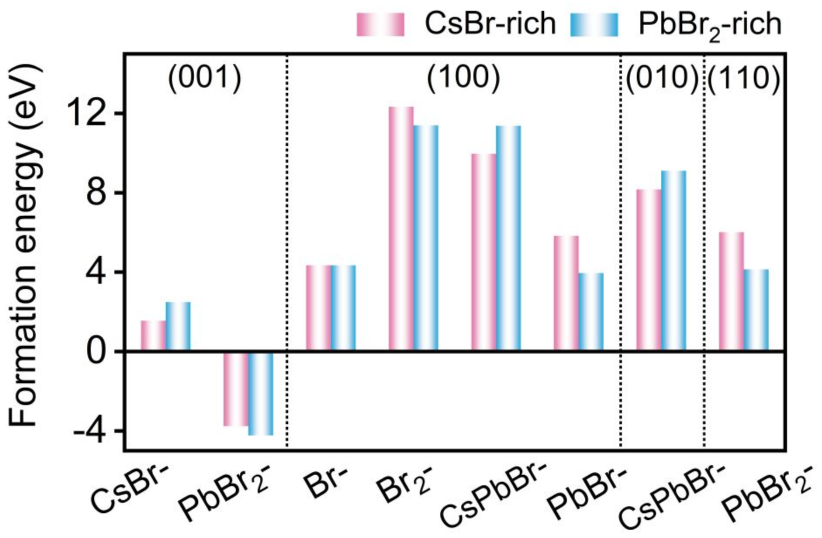

Figure S1. Defect formation energies of intrinsic $CsPbBr_3$ surfaces at neutral charge state under CsBr-rich and $PbBr_2$-rich growth conditions.

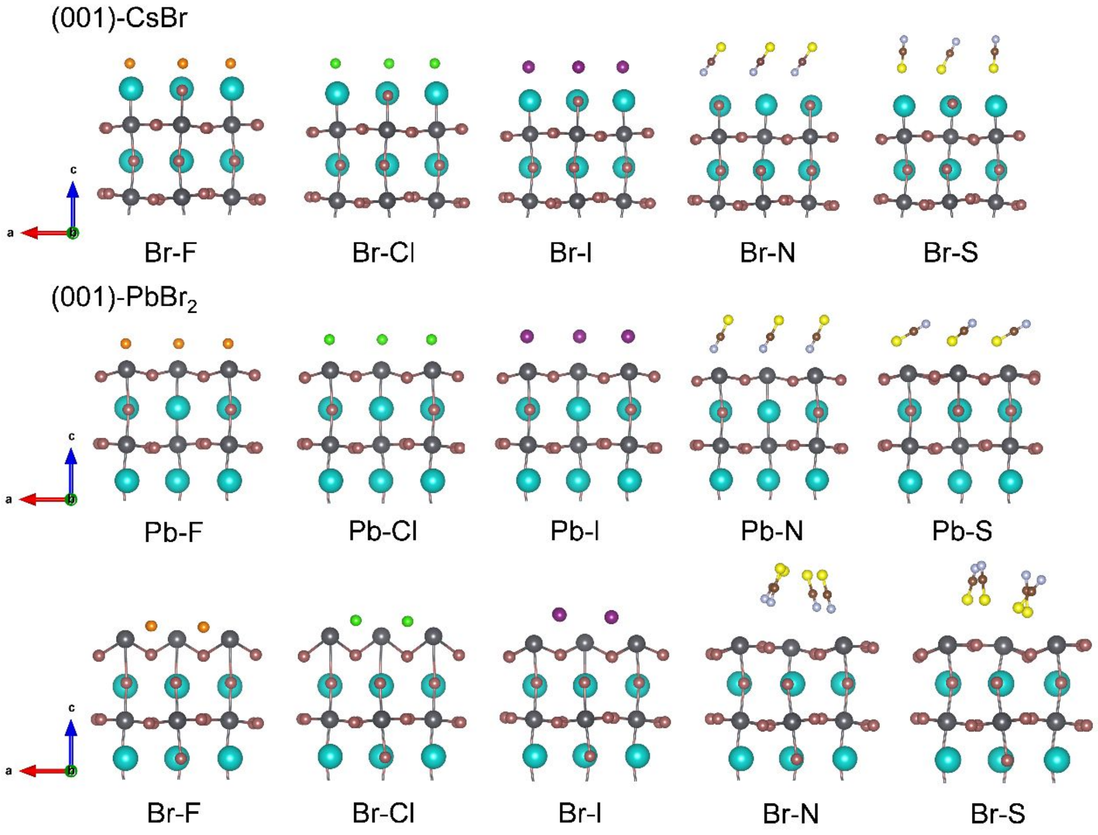

Figure S2. The relaxed atomic configurations of the top layer of $CsPbBr_3$ (001) surface after adsorption, including CsBr- and $PbBr_2$-terminations.

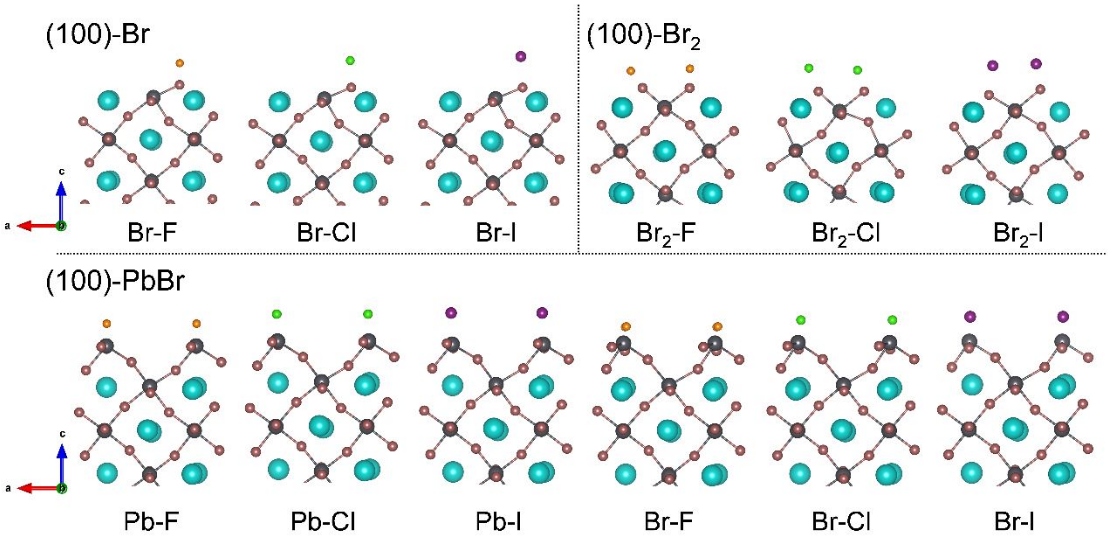

Figure S3. The relaxed structural models of the top layer of $CsPbBr_3$ (100) surface after adsorption, including Br-, Br_2- and PbBr-terminations.

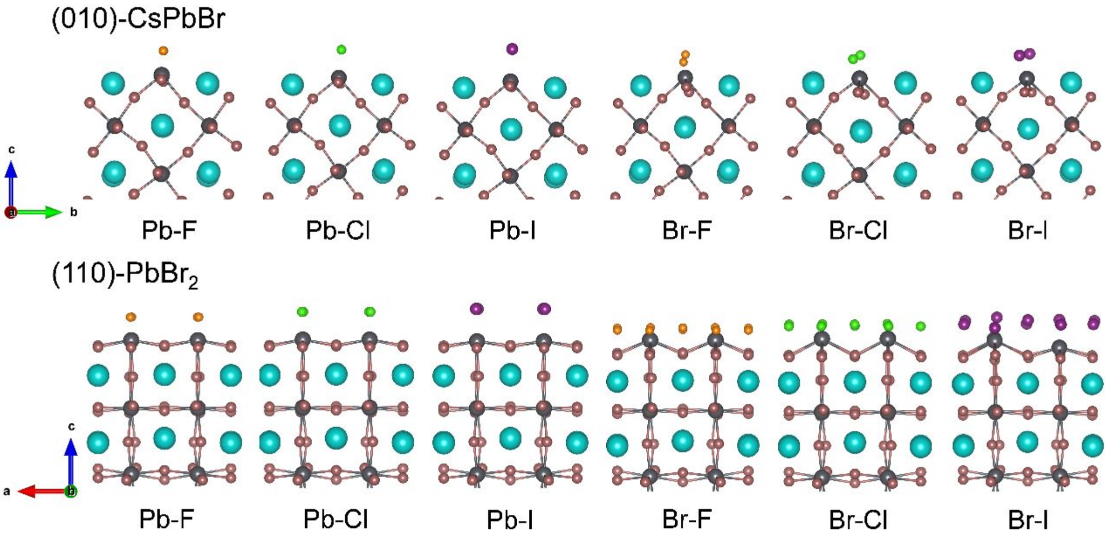

Figure S4. Top layer lattice structures of the (010) surface with CsPbBr termination and (110) surface with $PbBr_2$ termination after adsorption.

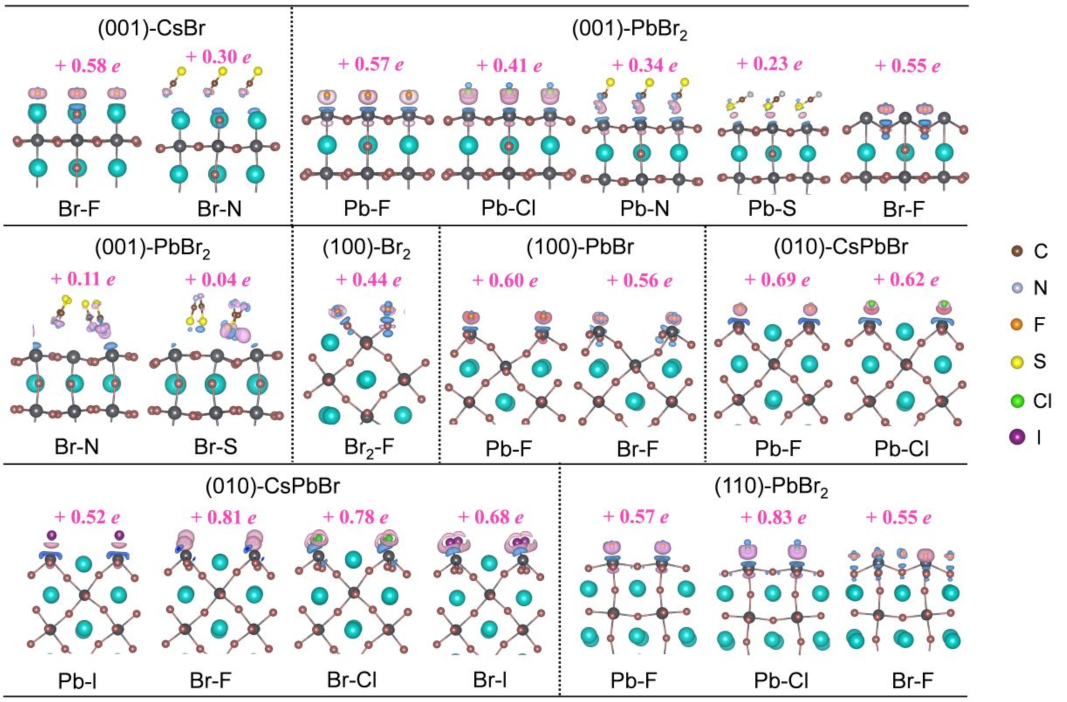

Figure S5. Charge density differences between adsorbates and $CsPbBr_3$ surfaces, along with specific amounts of transferred charge. Pink regions denote charge accumulation, while light blue areas signify charge depletion. The charge obtained of each adsorbed atom is shown above the top of each model.
